# A comprehensive review on privacy preserving data mining

**DOI:** 10.1186/s40064-015-1481-x

**Published:** 2015-11-12

**Authors:** Yousra Abdul Alsahib S. Aldeen, Mazleena Salleh, Mohammad Abdur Razzaque

**Affiliations:** Faculty of Computing, University Technology Malaysia, UTM, 81310 UTM Skudai, Johor Malaysia; Department of Computer Science, College of Education, Ibn Rushd, Baghdad University, Baghdad, Iraq

**Keywords:** Privacy preserving, Data mining, Distortion, Association, Classification, Clustering, Outsourcing, K-anonymity

## Abstract

Preservation of privacy in data mining has emerged as an absolute prerequisite for exchanging confidential information in terms of data analysis, validation, and publishing. Ever-escalating internet phishing posed severe threat on widespread propagation of sensitive information over the web. Conversely, the dubious feelings and contentions mediated unwillingness of various information providers towards the reliability protection of data from disclosure often results utter rejection in data sharing or incorrect information sharing. This article provides a panoramic overview on new perspective and systematic interpretation of a list published literatures via their meticulous organization in subcategories. The fundamental notions of the existing privacy preserving data mining methods, their merits, and shortcomings are presented. The current privacy preserving data mining techniques are classified based on distortion, association rule, hide association rule, taxonomy, clustering, associative classification, outsourced data mining, distributed, and k-anonymity, where their notable advantages and disadvantages are emphasized. This careful scrutiny reveals the past development, present research challenges, future trends, the gaps and weaknesses. Further significant enhancements for more robust privacy protection and preservation are affirmed to be mandatory.

## Background

Supreme cyberspace protection against internet phishing became a necessity. The intimidation imposed via ever-increasing phishing attacks with advanced deceptions created a new challenge in terms of mitigation. Lately, internet phishing caused significant security and economic concerns on the users and enterprises worldwide. Diversified communication channels via internet services such as electronic commerce, online-banking, research, and online trade exploiting both human and software vulnerabilities suffered from tremendous financial loss. Therefore, enhanced privacy preserving data mining methods are ever-demanding for secured and reliable information exchange over the internet. The dramatic increase of storing customers’ personal data led to an enhanced complexity of data mining algorithm with significant impact on the information sharing. Amongst several existing algorithm, the Privacy Preserving Data Mining (PPDM) renders excellent results related to inner perception of privacy preservation and data mining. Truly, the privacy must protect all the three mining aspects including association rules, classification, and clustering (Sachan et al. 2013). The problems faced in data mining are widely deliberated in many communities such as the database, the statistical disclosure control and the cryptography community (Nayak and Devi 2011). The emergence new cloud computing technology allowed the business collaborators to share the data and supply the information for the mutual benefits. All of these are related to the cumulative capability to store users’ individual data together with the rising complexity of data mining algorithms that affects the information exchange. Yet, the concepts, utilization, categorization, and various attributes of PPDM in terms of its strength and weakness are not methodically reviewed.

Currently, several privacy preservation methods for data mining are available. These include *K*-anonymity, classification, clustering, association rule, distributed privacy preservation, *L*-diverse, randomization, taxonomy tree, condensation, and cryptographic (Sachan et al. 2013). The PPDM methods protect the data by changing them to mask or erase the original sensitive one to be concealed. Typically, they are based on the concepts of privacy failure, the capacity to determine the original user data from the modified one, loss of information and estimation of the data accuracy loss (Xu and Yi 2011). The basic purpose of these approaches is to render a trade-off among accuracy and privacy. Other approaches that employ cryptographic techniques to prevent information leakage are computationally very expensive (Ciriani et al. 2008). Conversely, PPDMs use data distribution and horizontally or vertically distributed partitioning through multiple entities.

Sometimes the individuals are reluctant to share the entire data set and may wish to block the information using varieties of protocols. The main rationale for implementing such techniques is to maintain individuals’ privacy while deriving collective results over the entire data (Aggarwal and Yu 2008). Despite much research a method with satisfactory privacy settings are far from being achieved. It is essential to protect the data information before it gets distributed to multi-cloud providers. To protect the privacy, clients’ information must be identified prior to sharing with those unknown users not directly allowed to access the relevant data. This can be achieved by deleting from the dataset the unique identity fields such as name and passport number. Despite this information removal, there are still other types of information including date of birth, zip code, gender, number of child, number of calls, and account numbers which can be used for possible subjects’ identification. Intensified and extensively robust privacy preservation measures in data mining must be implemented to prevent such types of breaching.

This presentation underscores the significant development of privacy preserving data mining methods, the future vision and fundamental insight. Several perspectives and new elucidations on privacy preserving data mining approaches are rendered. Existing literatures are systematically subcategorized to identify the strengths, gap, and weakness of various approaches. The paper is organized as follows. “Privacy preserving data mining” discusses in detail the requirement of privacy preserving data mining scheme in the context of internet phishing mitigation. The notable advantages and disadvantages of the existing methods are highlighted in “Shortcomings of PPDM methods”. This section primarily focused on the creation of awareness and relevant action to be taken by all relevant quarters to protect privacy in secured data transfer over the web. “Conclusion” concludes the paper with further outlook in this field.

### Differential privacy model

Recently, differential privacy model is widely explored to render maximum security to the private statistical databases by minimizing the chances of records identification. There are several trusted party that holds a dataset of sensitive information such as medical records, voter registration information, email usage, and tourism. The primary aim is to providing global, statistical information about the data publicly available, while protecting those users privacy whose information is contained in the dataset. The concept of “indistinguishability” also called “differential privacy” signifies the “privacy” in the context of statistical databases. Generally, data privacy is viewed as a characteristic or annotation to data safety. Obviously, this view is incorrect because the objectives of the two domains are opposite. Conversely, security protects the data against unauthorized access when transmitted across a network. However, upon arriving to an authorized user no additional constraints are imposed on the data security to revealing the personal information of an individual. Thus, it is worth to determine the correlation between data security and data privacy because the former is prerequisite of the latter.

Data must be protected at storage and the transmission must be made via data security protocols. Moreover, in case data privacy is a goal, then some other steps must be considered to protect individuals confidentiality embodied in the data. It is important to describe the process of PPDM addresses in terms of data sharing and the results of data mining operation between a number of users *u*_*1*_*,…u*_*m*_ with *m* ≥ 2. The data is viewed as a database of *n* records, each consisting of *l* fields, where each record represents an individual *i*_*i*_ and illustrates them through its fields. In a simplified representation a table *T* contains rows to signify *i*_*1*_*,…i*_*n*_ and columns that symbolizes the fields *a*_*1*_*,…a*_*l*_. Assuming a fixed representation, each individual is represented by a vector of components *a*_*1*_*,…a*_*l*_. The most useful dimension in PPDM is the protected privacy embedded in *T,* which an attacker wants to acquire. The other practical dimension is the possessive data structure, which belongs to one entity and need to be shared with another (m = 2). It may be built from parts owned by different entities.

It is important to introduce some definitions to strengthen the PPDM concepts. Especially, an explicit identifier is an attribute that permits a direct connection of an instance (a row in T) to a user *i*. For example, by identifying a cellular phone number or a driver’s license number it may unambiguously connect the row in *T,* where this explicit identifier to a person *i* is embedded. Conversely, a quasi-identifier being a set of individuals’ non-explicit attributes may also link a row in *T* to a specific person. For instance, in the United States the quasi-identifier triplet <date of birth, 5 digit postal code, gender> uniquely identifies 87 % of the nation’s population (Sweeney 2002). By combining a public healthcare information dataset with a publicly available voters’ list and using quasi-identifiers, Sweeney convinced that it is possible to mine the secret health records of all state employees from a published dataset of the Massachusetts governor, where only explicit identifiers is removed. Generally, the primary PPDM identity protection methods that are drawn on simple ideas are known to people as they are abundantly accessible in the literatures and films. These concepts are portrayed as “hiding in the crowd” and “camouflage”. One of the “hiding in the crowd” approach to data privacy is the k-anonymity. Actually, the k-anonymity method (Sweeney 2002; Nergiz et al. 2009) modifies the original data *T* to obtain *T*′ such that for any quasi-identifier *q* that can be built from attributes of *T* there are at least *k* instances in *T*′ so that q matches these instances. Moreover, datasets require generalization to satisfy *k*-anonymity.

## Privacy preserving data mining

Recently, the relevance of privacy-preserving data mining techniques is thoroughly analyzed and discussed by Matwin (2013). Utilization of specific methods revealed their ability to preventing the discriminatory use of data mining. Some methods suggested that any stigmatized group must not be targeted more on generalization of data than the general population. Vatsalan et al. (2013) reviewed the technique called ‘Privacy-Preserving Record Linkage’ (PPRL), which allowed the linkage of databases to organizations by protecting the privacy. Thus, a PPRL methods based taxonomy is proposed to analyse them in 15 dimensions. Qi and Zong (2012) overviewed several available techniques of data mining for the privacy protection depending on data distribution, distortion, mining algorithms, and data or rules hiding. Regarding data distribution, only few algorithms are currently used for privacy protection data mining on centralized and distributed data. Raju et al. (2009) acknowledged the need to add or to multiply the protocol based homomorphic encryption along with the existing concept of digital envelope technique in obtaining collaborative data mining while keeping the private data intact among the mutual parties. The proposed technique exhibited considerable influence on different applications.

Malina and Hajny (2013) and Sachan et al. (2013) analysed the current privacy preserving solutions for cloud services, where the solution is outlined based on advanced cryptographic components. The solution offered the anonymous access, the unlink ability and the retention of confidentiality of transmitted data. Finally, this solution is implemented, the experimental results are obtained and the performance is compared. Mukkamala and Ashok (2011) compared a set of fuzzy-based mapping methods in the context of privacy-preserving characteristics and the capability to maintain the same connection with other fields. This comparison is subjected to: (1) the four front modification of the fuzzy function definition, (2) the introduction of the seven ways to join different functional values of a particular data item to a single value, (3) the utilization of several similarity metrics for the comparison of the original data and mapped data, and (4) the evaluation of the influence of mapping on the derived association rule.

### Data distortion dependent PPDM

Kamakshi (2012) proposed a novel idea to dynamically identify the sensitive attributes of PPDM. Identification of these attributes depends on the threshold limit of sensitivity of each characteristic. It is observed that the data owner modified the value under identified sensitive attributes using swapping technique to protect the privacy of sensitive information. The data is modified in such a manner that the original properties of the data remain unchanged. Despite the novelty it remains time expensive. Subsequently, Zhang et al. (2012a) introduced a newly enhanced historical probability based noise generation strategy called HPNGS. The simulation results confirmed that the HPNGS is capable in reducing the number of noise requirements over its random complement as much as 90 %. Later, they focused on the privacy protection and noise obfuscation in cloud computing (Zhang et al. 2012b). Consequently, a novel association probability based noise generation strategy (APNGS) is developed. The analysis confirmed that the proposed APNGS significantly improved the privacy protection on noise obfuscation involving association probabilities at a reasonable extra cost than standard representative strategies.

Li et al. (2009a) presented a low-cost and less risky anonymous perturbation technique via homomorphism encryption and anonymous exchange. The proposed technique displayed robustness for optimized parameters. It is complex, loss in utility of data. Kamakshi and Babu (2010) introduced three models including clients, data centres, and database in every site. The data centre is completely passive, so that the clients and the site database role appear exchangeable. Islam and Brankovic (2011) proposed an architecture involving different novel techniques that affected all the attributes in the database. Experimental findings showed that the proposed architecture is very efficient in preserving the original patterns in a perturbed dataset. Wang and Lee (2008) introduced a technique to prevent Forward-Inference Attacks, in the sanitized data (implies original data) created by the sanitization.

### Association rule based PPDM

An improved distortion technique for privacy preserving frequent item-set mining is proposed by Shrivastava et al. (2011), where two probability parameters (fp and nfp) are employed. Better accuracy is achieved in the presence of a minor reduction in the privacy by tuning these two parameters. Furthermore, this algorithm produced the optimum results when the fraction of frequent items among all the available items is less. PPDM is used in various fields for its enhanced efficiency and security. Presently, it is facing a rule mining challenge. Vijayarani et al. (2010a) explained the techniques of statistical disclosure control community, the database community, and the cryptography community. Less utility of data requires high cost. Aggarwal and Yu (2008) emphasized two significant factors involving the association rule mining such as confidence and support. For an association rule X => Y, the support is the percentage of transactions in the dataset which includes X U Y. The confidence (also called strength) of an association rule X => Y is the ratio of the transactions number by X. Furthermore, Belwal et al. (2013) reduced the basis of support and confidence of sensitive rules without modifying directly the given database. However, alteration can indirectly be performed via newly incorporating parameters associated to database transactions and association rules. New additions include M support (modified support), M confidence (modified confidence) and Hiding counter. The algorithm utilized the definition of support and confidence. Thus, it hided the required sensitive association rule without any side effect. However, it can hide only the rules for single sensitive item on the LHS.

Jain et al. (2011) developed a new algorithm to enhance and reduce the support of the LHS and RHS rule item to hide or secure the association rules. The proposed algorithm is found to be advantageous as it made minimum modification to the data entries to hide a set of rules with lesser CPU time than the previous work. It is limited to association rule only. Naeem et al. (2010) proposed an architecture which screened the restricted association rules with complete removal of the known side effects such as the generation of unwanted, non-genuine association rules while yielding no ‘hiding’ failure. In this architecture, standard statistical measures are used instead of conventional framework of support and confidence to create association rules, particularly weighing procedure based on central tendency. Li and Liu (2009) introduced an association rule mining algorithm for privacy preserving known as DDIL. The proposed algorithm is based on inquiry limitation and data disturbance. The original data can be hidden or disturbed by using DDIL algorithm to improve the privacy efficiently. This is an effective technique to generating frequent items from transformed data. Experimental results displayed that the proposed technique is efficient to generating acceptable values of privacy balance with suitable selection of random parameters.

### Hide association rule based PPDM

Fast Hiding Sensitive Association Rules (FHSAR) algorithm is introduced by Weng et al. (2008). This secured the SAR with fewer side effects, where a strategy is established to avoid hidden failures. Besides, two heuristic techniques are developed to improve the efficiency of the system to solve the problems. The heuristic function is further utilized to determine the earlier weight for each particular transaction so that the order of modified transactions can be decided efficiently. Consequently, the connection between the sensitive association rules and each transaction in the original database are analyzed by successfully choosing the suitable item for modification. The efficient sanitization of sensitive information for updated database need to be studied. Dehkordi et al. (2009) presented a new multi-objective technique to hide the sensitive association rules and to enhance the security of database. In fact, this maintained the utility and of mined rules at efficient level. The proposed algorithm is based on genetic algorithm (GA) concept, where the privacy and accuracy of dataset are enhanced. Gkoulalas-Divanis and Verykios (2009) developed an exact border-based technique to obtain an optimal solution to hide sensitive frequent item sets with minimum extension of the original database generated synthetically via the database extension. This is accomplished via the following: (1) by formulating the generation of the database extension as a constraint satisfaction problem, (2) using mapping of the constraint satisfaction issues to an equivalent binary integer programming problem, (3) via the manipulation of underutilized synthetic transactions to increase the support of non-sensitive item sets, (4) employing the minimally relaxing constraint satisfaction problem to offer an approximate solution close to the optimal one when an ideal solution does not exist, and (5) by partitioning the universe of the items to enhance the efficiency of the proposed hiding algorithm.

Li et al. (2009b) proposed a new algorithm to sanitize a transactional database. This is item-set oriented, where the support of large item-sets are considerably reduced below the threshold defined by the client. Thus, no rules can be obtained from the specific item-sets. A new technique is also introduced to select the items that required removal from the dataset to avoid the detection of a set of rules. The main limitations are associated with the selection of victim-items without affecting the non-sensitive patterns when the sanitization of 3rd and the 4th sensitive transactions are defined. Kasthuri and Meyyappan (2013) presented a new technique to identify the sensitive items by hiding the susceptible association rules. The proposed technique located the frequent item sets and produced the association rules. Representative association rules concept is employed to detect the sensitive items. Hiding the sensitive association rules using selected sensitive items is worth looking. Quoc et al. (2013) have developed heuristic algorithm based on the intersection lattice of frequent item-sets to secure the set of sensitive association rules employing distortion method. To reduce the side effects, the heuristic for confidence and support reduction based on intersection lattice (HCSRIL) algorithm are used. This specified the victim item and reduced the number of transactions by causing least impact on item-sets variations in Gen(FI). In addition, Domadiya and Rao (2013) introduced a heuristic based algorithm called Modified Decrease Support of RHS item of Rule Clusters (MDSRRC) to secure the delicate association rules using multiple items in consequent (RHS) and antecedent (LHS). This algorithm successfully addressed the drawbacks of existing rule hiding DSRRC algorithm. Experimental findings revealed the efficiency and capability of the proposed algorithm to maintaining the database quality. By minimizing the modifications on database the efficiency can be enhanced with reduced side effects.

### Classification based PPDM

Xiong et al. (2006) proposed a closet neighbour classification method based on SMC techniques to resolve the privacy challenges in few stages including the pf selection of the privacy preserving closet neighbour and the categorization of privacy preserving. The proposed algorithm is balanced in terms of accuracy, performance, and privacy protection. Furthermore, it is adaptable to the various settings to fulfilling different optimization condition. Singh et al. (2010) provided a simple and efficient privacy preserving classification for cloud data. Jaccard similarity measure is used to compute the nearest neighbours for *K*-NN classification and the equality test is introduced to compute it between two encrypted records. This approach facilitated a secured local neighbour computation at each node in the cloud and classified the unseen records via weighted *K*-NN classification scheme. It is significant to focus on enabling the robustness of the presented approach so that generalization to multiple data mining tasks can be made, where security and privacy are needed.

Baotou (2010) introduced an efficient algorithm based on random perturbation matrix to protect privacy classification mining. It is applied on discrete data of character type, Boolean type, classification type and number types. The experimental revealed the significantly enhanced features of proposed algorithm in terms of privacy protection and accuracy of mining computation, where the computation process is greatly simplified but at higher cost. Vaidya et al. (2008) developed an approach for vertically partitioned mining data. This technique could modify and extend a variety of data mining applications as decision trees. More efficient solutions are needed to find tight upper bound on the complexity. Kantarcıoglu and Vaidya (2003) emphasized the use of secure logarithm and summation, where the distributed naive Bayes classifier are securely determined. The experimental results strongly supported the concept of few useful protected protocols that facilitated the secure deployment of different types of distributed data mining algorithms. The classification of privacy preserving methods and standard algorithms for each class is reviewed by Sathiyapriya and Sadasivam (2013), where the merits and limitations of different methods are exemplified. The optimal sanitization is found to be NP-Hard in the presence of privacy and accuracy trade-off.

### Clustering based PPDM

Yi and Zhang (2013) overviewed various earlier solutions to preserve privacy of distributed k-means clustering and provided a formal definition for equally contributed multiparty protocol. An equally contributed multiparty k-means clustering is applied on vertically partitioned data, wherein each data site contributed k-means clustering evenly. According to basic concept, data sites collaborated to encrypt k values (each associated to a distance between the centre and point) with a common public key in each step of clustering. Then, it securely compared k values and outputted the index of the minimum without displaying the intermediate values. In some setting, this is practical and more efficient than Vaidya–Clifton protocol (Vaidya et al. 2008).

### Associative classification based PPDM

An associative classification model based on vertically partitioned datasets is introduced by Raghuram and Gyani (2012). A scalar product based third party privacy preserving model is adopted to preserve the privacy for data sharing process between multiple users. The accuracy of the presented method is authenticated on its VCI databases with inspiring results. Lin and Lo (2013) presented a set of algorithms comprising of Equal Working Set (EWS), Small Size Working Set (SSWS), Request on Demand (ROD) and the Progressive Size Working Set (PSWS). This repeated mining offered a scalable, fast and reliable service for different-tasks on computing environments. The presented algorithms demonstrated an outstanding efficiency in terms of scalability and execution time under different simulation conditions. Although CARM is a fast and scalable distributed algorithm in comparison with previous studies, the scalability is still limited. This is because the HD-Mine used in CARM establishes the FP-tree in the main memory of the trusted node. In the absence of any memory space to mine the conditional FP-tree in the trusted node, the reconstructed conditional FP-tree is distributed to an available computing node for mining. The trusted node must provide sufficient memory space for the original FP-tree. Clearly, the scalability is restricted by the major memory size of the trusted node.

Harnsamut and Natwichai (2008) developed a novel heuristic algorithm based on Classification Correction Rate (CCR) of particular database to secure the privacy and sustain the quality of data. The proposed algorithm is tested and the experimental results are validated. The heuristic algorithm is found to be highly effective and efficient. Seisungsittisunti and Natwichai 2011) highlighted the issues related to data transformation to protecting privacy for data mining technique and associative classification in an incremental-data scenario. An incremental polynomial-time algorithm is proposed to transform the data to maintain a privacy standard called k-anonymity. Quality can still be maintained even under transformation when constructing an associative classification model. Different experiments are performed to evaluate developed algorithm performance and compared with non-incremental algorithm. It is established to be more efficient in every problem setting. It is worth to examine the stored data in the distributed systems rather than a single repository.

### Privacy preserving outsourced data mining

Giannotti et al. (2013) explained the issues involving the outsourcing of association rule mining task for a corporate privacy-preserving network. An attack model is developed based on the background knowledge for privacy preserving outsourced mining. An encryption scheme, known as Rob Frugal is proposed. This is based on 1–1 substitution ciphers of items, which included the fake transactions to share each cipher item with the same frequency as ≥k − 1 to the others. A compact synopsis of the fake transactions is used for true support of mined patterns from which the server can be recovered efficiently. It is demonstrated that the proposed scheme is robust against adversarial attack which is based on the actual items and their exact support. This framework assumed that the attacker is unaware of such information. Furthermore, any relaxation may break our encryption scheme and bring privacy vulnerabilities. They investigated encryption schemes that could resist such privacy vulnerabilities. The strategies for the improvement of the RobFrugal algorithm to minimize the number of spurious patterns are also explored.

Worku et al. (2014) enhanced efficiency of the above scheme by reducing the computational intensive operations such as bilinear mapping. The scheme revealed secure and efficient results after a detailed analysis on security performance. However, the data block insertion made the proposed scheme non-dynamic. Thus, the development of a fully dynamic and secure public auditing scheme remains an open challenge for a cloud system. Arunadevi and Anuradha (2014) investigated the issues related to outsourcing of frequent item-sets for a corporate privacy preserving architecture. An attack model is introduced by considering that the attackers are fully aware of the items and support of the item. In addition, even in the eventuality the attackers are totally conscious of the details of the encryption algorithm and some pairs of item with the corresponding cipher values. These basic assumptions remarkably improved the security of the system and eliminated the item and item-set based attack as well as reduced the processing time.

Lai et al. (2014) proposed the first semantically secured solution for outsourcing association rule mining with data privacy, mining privacy and soundness. These solutions are non-deterministic and secured against an adversary at cloud servers. It is capable to adaptively obtaining plaintext–cipher text pairs as required by semantic security. The adversary may also insert false data into the data mining results. In comparison, adversary models used in previous works on outsourcing association rule mining assumed that the honesty of adversary/server but remained curious. It is not capable to obtaining any plaintext–cipher text pairs in attacks. Consequently, the sub-situation mappings based solutions are neither semantically secured nor ensured the soundness for the data mining results. Kerschbaum and Julien (2008) presented a searchable encryption scheme for outsource data analysis. In this scheme the client had to encrypt the data only once and transmit the encrypted information to the data analyst. The data analyst conducted a number of queries for required permission from the client to translate the data contents in the queries. The proposed encryption schemes permitted the search of keyword and range queries. The scheme also allowed queries to reprocess the output of earlier queries as tokens to make dependent queries without interface. The proposed scheme is found to be secured. There are many open questions in the area of search-able encryption. In case of outsourced data analytics, it is most interesting to combine the efficiency improvements possible for range queries with the necessary security requirements via pairing-based cryptography.

### Distributed method based PPDM

Ying-hua et al. (2011) surveyed the Distributed Privacy Preserving Data Mining (DPPDM) depending on different underlying technologies. Existing techniques are categorized into three groups such as (1) secure multi-party computation, (2) perturbation and (3) restricted query. Li (2013) elucidated the advantages and drawbacks of each method by developing and analyzing a symmetric-key based privacy-preserving scheme to support mining counts. An incentive consideration is proposed to the study the secure computation by presenting a reputation system in wireless network. The proposed system offered an incentive for misbehaving nodes to behave properly. Experimental results revealed the system effectiveness in detecting the misbehaving nodes and enhancing the average throughput in the whole network. Furthermore, Dev et al. 2012) acknowledged the privacy risks related to data mining on cloud system and presented a distributed framework to remove such risks. The proposed approach involved classification, disintegration, and distribution. This avoided the data mining by preserving the privacy levels, splitting the data into chunks and storing them into suitable cloud providers. Though, the proposed system offered a suitable way to safe privacy from mining based attacks, but it added a performance overhead as client accessed the data frequently. For instance, client had to run a global data analysis for a complete dataset, where the analysis required accessing the data through different locations with a degraded performance.

Tassa (2014) developed a protocol for secured mining of association rules in horizontally distributed database. The proposed protocol possessed advantages over leading protocols in terms of performance and security. It included two set of rules including (1) a multi-party protocol to compute the union or intersection of private subsets possessed by each client and (2) a protocol to test the presence of an element held by client in a subset held by another. Techniques based on Field and Row-Level distribution of transactional data are proposed by Chan and Keng (2013). They presented a distributed framework to preserve outsourcing association mining rules and explored the possibility of its deployment. Database information based on its characteristics is distinguished for the distribution to multiple servers. Its privacy notions are examined from two separate viewpoints such as distribution of support values and K-anonymity. The proposed algorithms for allocating transactions to outsourced servers are based on the importance of the types of privacy notion to a user. Dong and Kresman (2009) explained the relation between distributed data mining and prevention of indirect disclosure of private data in privacy preserving algorithms, where two protocols are devised to avoid such disclosures. The first one was a simple add-on to a protocol used for different application, whereas the second one provided the suitability of collusion resistance and fewer broadcasts. The simplicity of the proposed protocols enabled minimal requirements for computation, easy data storage or data structures. Consequently, the notion of trust is introduced and the performance of certain ID assignment protocols is addressed.

Aggarwal et al. (2005) discussed data encryption based methods, which caused a large overhead in query processing. A new distributed framework is proposed to enable privacy-preservation for the outsourced storage of data. Different techniques are used to decompose the data. It demonstrated improved queries when implemented in such types of distributed system. A new definition for privacy is coined based on hiding sets of attributes. It discussed the secured privacy achievement of the proposed decomposition approaches and identified the best privacy-preserving decomposition technique. Other future work includes identifying improved algorithms for decomposition, expanding the scope of techniques available for decomposition (supporting replication, and incorporation of these techniques into the query optimization framework). Xu and Yi (2011) investigated the privacy-preserving distributed data mining that passed through different stages and persisted. Taxonomy is proposed to endorse the standardization and assessment of the protocols efficiency. This might be applied to categorize such PPDDM protocols based on predefined dimensions. The dimensions included the data partitioning model, mining algorithms, privacy preservation methods and secured communication model. This area is prospective. Yet, the solution and evaluation work is still open for further investigation.

Inan and Saygin (2010) presented a technique to assemble dissimilarity matrix for horizontal distributed data mining. The comparison required all the record operations in the form of pair for personal private datasets which are distributed horizontally to different sites. This approach considered the data either in the form of character or numerical. For these two different types of data sets, a number of comparison functions are made available. However, as expected, ensuring privacy has its costs, considering the comparison against the baseline protocol where private data is shared with third parties. We used the secured comparison protocols for clustering horizontally partitioned datasets. There are various other application areas of these methods such as record linkage and outlier detection problems Nanavati and Jinwala (2012) elaborated different approaches used to find global and partial cycles in a distributed setup while keeping the privacy of the particular parties secured in a co-operative setup. The interleaved algorithm is extended and modified to determine global cycles in cyclic association rules privately. The privacy preservation techniques are recommended on the basis of homomorphic approach and secret sharing. It is concluded that the approaches based on Shamir’s secret sharing can be employed to detect the partial global cycles. However, few open research challenges including the application of these privacy preserving theories to other temporal rule mining methods like calendric association rules and temporal predicate association rules need to be addressed. Another research challenge also involves deciphering the most efficient and accurate technique in this scenario by practically comparing the cost for each method.

Agrawal and Srikant (2000) developed a uniform randomization method based association rule for the categorical datasets. In this approach, before sending a data to server, the client is replaced each item by a new item which is originally absent in the data. The substitution process of specific values from datasets with other values is called uniform randomization. This is a generalization of the Warner’s “randomized response” technique. In other types of data reconstruction techniques the original data are put aside and are initiated via sanitizing known as “knowledge base”. Thus, newly obtained data is then reassembled based on the sanitized knowledge. The effectiveness of randomization with reconstruction for categorical attributes is exemplified.

Wang et al. (2010) proposed a modified algorithm called PPFDM and related computation technique based on the Frequent Data Mining (FDM) to preserve privacy. The process involved the computation of total support count along with the privacy-preserved technique while ensuring the local large item-set and local support count source is covered. Thus, the time needed for the communication is saved and secured the distributed data privacy at each site. The experimental results demonstrated the effectiveness and suitability of the method for practical application, especially in privacy preservation during mining process.

Nguyen et al. (2012) presented an Enhanced M. Hussein et al.’s Scheme (EMHS) for secured privacy association rules mining, where horizontally distributed database is used. EMHS (developed in 2008) is capable to modify the privacy and efficiency with increasing number of sites. The efficiency of EMHS is discerned to be much better than MHS, particularly for databases with increasing number of sites. A second approach is also presented for the other types of datasets. It is important to solving the collusion of Initiator and Combiner. Om Kumar et al. (2013) used WEKA to predict the patterns in a single cloud. By using cloud data distributor with a secured distributed approach they provided an effective solution that prevented such mining attacks on cloud. Thus, it made the cloud a secured platform for service and storage.

Mokeddem and Belbachir (2010) proposed a distributed model to perform class-association rules detection for shared-nothing framework. The solution of the proposed model is one of the fastest known sequential algorithms (FP-growth) which is extended to produce classification rules in a parallel setting. By using the proposed system, the data replication is avoided on these sites with an option to communicate the required information. These choices are evaluated by performing experimentations, which permitted us to analyze several important aspects such as accuracy, scalability, speedup, memory usage, communication, synchronization, and also the load balancing. Ibrahim et al. (2012) developed a practical cryptographic model to calculate the KNN categorization over the distributed cloud databases. Their experiments demonstrated similar accuracy of the proposed as the naive scheme without security. It is believed that such schemes may mitigate the users concerns and accelerate the paces towards the high adoption of cloud computing. The extension of our secure classifier to work in the malicious adversary security model will be reported elsewhere.

Patel et al. (2012) proposed an operative algorithm to protect the secrecy distributed over K-Means cluster using Shamir’s secret sharing model. The proposed approach computed the cluster mean collaboratively and prevented the role of trusted third party. Upon comparison, it is observed that the proposed framework is orders of magnitude faster as compared to oblivious polynomial evaluation and homomorphic encryption techniques in terms of computation cost and more reliable for huge databases. It is essential to extend the proposed algorithm in vertical partitioning in the presence of malicious adversary model. In addition, the results from a realistic distributed emulation are worth looking. Kumbhar and Kharat (2012) analysed and compared different techniques used for Privacy Preserving Association Rule Mining (PPARM). The algorithm based on cryptography techniques, Homomorphic encryption, Secure Scalar product and Shamir’s secret sharing technique are employed to satisfy the privacy constraints for vertically partitioned dataset. However, for horizontally partitioned dataset the algorithm with the combination of RSA public key cryptosystem and Homomorphic encryption scheme are used. Paillier cryptosystem is employed to determine the global supports. In practice, while calculating c.count collaboratively, participant may deviate from algorithm and lead malicious behaviour. But algorithm is semantically secured and prevents collusive behaviour with accurate results.

Nix et al. (2012) implemented two sketching protocols for the scalar (dot) product of two vectors which are used as sub-protocols in larger data mining tasks. Results through extensive experimentations revealed their high accuracy, low data leakage, and orders of magnitude improved efficiency. The security properties of these approximations under a security definition are also analyzed. In contrast to the previous definitions these are found to be very efficient approximation protocols. It is worth to explore the use of these dot product protocols in other data mining tasks such as support vector machines, neural networks, and clustering. The notion of a secure approximation and determination of the relaxation extent of the posed restrictions by the security model need to be looked at.

Keshavamurthy et al. (2013) demonstrated that GA approach possessed two potential advantages than traditional frequent pattern mining algorithm. It is found that in frequent pattern mining, the population is formed only once. Conversely, in GA method the population is formed for each generation that maximizes the sample set. However, the major drawback of GA approach is connected to the duplication in its sequential generations. For privacy preservation data mining over distributed dataset, the key goal is to permit computation of collective statistics for complete database with assurance of the privacy for confidential data of the contributing databases. Hence, the algorithms for privacy preservation needs further improvement based on the trade-offs between reconstruction accuracy and privacy. On top, the fitness function of GA plays an important role and the convergence of search space is directly proportional to the effectiveness of fitness function. In other words, superior fitness functions for a given problem leads to faster convergence of GA.

### K-anonymity based PPDM

For the sake of clarity, it is customary to render two important definition of K-anonymity.

The first definition tells that: *QI* being a quasi-identifier for a given table *U* with $T(A_{1} \ldots A_{n} )$, ${\text{f}}_{C} :U \to T,\;f_{g} :T \to U^{{\prime }}$, where $U \subseteq U^{{\prime }}$, a quasi-identifier of $T(Q_{T} )$ is a set of attributes $\{ Ai \ldots A_{J} \} \subseteq \{ {\text{A}}_{1} \ldots {\text{A}}_{n} \}$, where $\exists p_{i} \in U$*such that*$f_{g} \,(f_{c} (p_{i} )[Q_{T} ]) = p_{i}$ (Sweeney 2002). The second definition is stated as follows: a table *T* satisfies *K*-anonymity if for every tuple ${\text{t}} \in {\text{T}}$ there exist *k* − *1* other tuples $t_{i1} t_{i2} \ldots t_{k - 1} \in T$ such that $t_{i1} [{\text{C}}] = t_{i2} [{\text{C}}] = \ldots t_{ik - 1} [{\text{C}}]$ for all ${\text{C}} \in {\text{Q}}_{\text{T}}$ (Machanavajjhala et al. 2007).

A scalable solution for each repetition can examine at least one generalization for each attribute involved in the linking. (Wang et al. 2004) studied the data mining as a approach used for data masking called data mining based on privacy protection. The data mining methods are inspected in terms of data generalization concept, where the data mining is performed by hiding the original information instead of trends and patterns. After data masking, the common data mining methods are employed without any modification. Two key factors, quality and scalability are specifically focused. The quality issue is settled via the trade-off between privacy and information. The scalability issue is established employing new data architecture while focusing on good generalizations. Loukides and Gkoulalas-divanis (2012) proposed a novel technique to anonymize the data by satisfying the data publishers’ utilization necessities experiencing low information loss. An accurate information loss measure and an effective anonymization algorithm are introduced to minimize the information losses. Experimental investigations on click-stream and medical data revealed that that the proposed technique allowed more reliable query answers than the state state-of-the-art techniques which are equivalent in terms of efficiency. This work opens up several promising avenues for future research. These include examining how UAR can be extended to guard against both identity and sensitive information disclosure and how to produce anonymized data with guaranteed utility in certain data mining tasks, such as classification and association rule mining. Friedman et al. (2008) extended the definitions of K-anonymity to prove that the data mining model does not violate the K-anonymity of the clients represented in the learning examples. A tool is provided to determine the amount of anonymity retained during data mining. The proposed approach showed its employment capability to different data mining problems including classification, association rule mining and clustering.

The K-anonymity is further combined with data mining approach to protect the respondent’s identity. Ciriani et al. (2008) highlighted the potential threats to K-anonymity, which are raised via the implementation of mining to collect data and analyses of two main techniques to join K- anonymity in data mining. The different approaches employed to detect K-anonymity violations are also described. Subsequently, the elimination of these approaches in association rule mining and classification mining are emphasized. He et al. (2011) proposed an algorithm based on clustering to produce a utility-friendly anonymized version of micro data. This method is found to outperform the non-homogeneous technique where the size of QI-attribute is greater than 3. They achieved a clustering-based K-anonymity algorithm, which revealed considerable improvement in the utility performance when applied to several real datasets. Recently, K-anonymous privacy preservation is widely employed. Further modification appeared to be increasingly difficult without resolving several issues. Patil and Patankar (2013) examined the standard K-anonymity techniques and its applications. Some of the multidimensional K-anonymous investigation is carried out. Yet, the present are multidimensional data sets based K-anonymity algorithms using nearest neighbour strategy are useful to enhancing the quality of anonymity and reducing the information loss.

Lately, K-anonymity became one of the most important topics for privacy preservation. This can effectively avoid privacy leaks due to link attacks. Certainly, K-anonymity is one of the widely used approach in all fields (Zhu and Chen 2012). Soodejani et al. (2012) employed a version of the chase termed as standard chase, which put some restrictions on the dependencies and constrains, such as being positive and conjunctive. This area is prospective for future study in fathering investigations on the applicability of other versions of the chase in the method. The anonymity principle of their method reveals some similarities to the L-diversity privacy model. Investigation of other privacy models such as t-closeness may provide a stronger privacy model for the proposed method with extreme usefulness. Karim et al. (2012) proposed a numerical method to mine maximal frequent patterns with privacy preserving capability. This method showed an efficient data transformation technique, a novel encoded and compressed lattice structure and MFPM algorithm. The proposed lattice structure and MFPM algorithm reduced both the search space as well as the searching time. The experimental results displayed that the MFPM algorithm outperformed PC_Miner and existing maximal frequent pattern mining algorithms. Besides the lattice structure, it outperformed FP-like tree and PC_tree algorithm as well.

Loukides et al. (2012) proposed a rule-based privacy model that allowed data publishers to express fine-grained protection requirements for both identity and sensitive information disclosure. Based on this model, they developed two anonymization algorithms. Their first algorithm worked in a top-down fashion, employing an efficient strategy to recursively generalize data with low information loss. Conversely, the second algorithm used sampling and a mixture of bottom-up and top-down generalized heuristics. This greatly improved the scalability and maintained low information loss. Extensive experimentations show that these algorithms significantly outperformed the state-of-the-art in context of recalling data utilization, while keeping good protection and scalability. It provides a foundation for some future studies. First, while identity and sensitive information disclosure are the main concerns in data publishing, it is worth examining membership disclosure, in which inferring whether an individual’s record is contained in the published data is to be prevented. Second, it is worth to extend the proposed approach to anonymize disk-resident data with small memory consumption and I/O overhead.

Vijayarani et al. (2010b) studied K-anonymity as an interesting approach to protect micro data related to public or semi-public sectors from linking attacks. The possible threats to K-anonymity approach is described in detail. Particularly, the problems related to data and the approaches are identified to combine K-anonymity in data mining. Nergiz et al. (2009) improved and extended the definitions of K-anonymity to manifold relations definitions of K-anonymity expression. It is shown that earlier developed techniques either failed to secure privacy or as a whole reduced the data utilization, and data protection in a multiple relations setting. A new clustering algorithms is introduced to obtain multi-relational anonymity. Experimental results illustrated that the proposed technique is an effective approach in terms of utility and efficiency. Support for arbitrary schemes with multiple private entities must be considered.

The problem of secured outsourcing of frequent itemset mining on the multi-cloud environments is studied by Tai et al. (2013). Concerning the challenges in big data analysis, they suggested to partition the data into several parts and outsourced each part independently to different cloud based on pseudo-taxonomy, anonymization technique, known as KAT. They proposed DKNT to ensure the privacy security for each partial data outsourced to different clouds. Experimental results demonstrated excellent achievement in terms of protection and better computation efficiency as compared to those on a single machine. Tai et al. (2010) presented K-support anonymity, which provided protection against a knowledgeable attacker with the exact support information. To achieve the K-support anonymity, a pseudo taxonomy tree is introduced with the third party mine for the generalized frequent item-sets. The construction of the pseudo taxonomy tree facilitated the hiding of the original items and limited the fake items introduced in the encrypted database. The results showed very good privacy protection with moderate storage overhead. K-anonymity is further enhanced and improved by Pan et al. (2012). They analyzed and compared the developed K-anonymity models and discussed their applications. The modified K-anonymity models such as the L-diversity, (α, K)-anonymity and (α, L)-diversification K-anonymity overcome the existing limitations related to privacy. Few K-anonymous methods are employed in obtaining the main technology.

Based on suppression, Deivanai et al. proposed a new K-anonymity technique called ‘kactus’ (Deivanai et al. 2011). In the proposed technique, multi-dimensional suppression is performed. The values are suppressed to a certain records based on other attributes without using the domain hierarchy trees. Thus, this approach identified the attributes independent of classification of the data records and suppressed these values to comply with K-anonymity. This approach is implemented on different database to determine its accuracy and efficiency and compared with other K-anonymity based techniques. It is affirmed that in a multiparty environment, the anonymization can be performed with perturbation to preserve privacy. A new definition of K-anonymity model for effective privacy protection of personal sequential data is introduced (Monreale et al. 2014). This method transformed the sequential datasets into a K-anonymous form, while preserving the utility of data with reference to a variety of analytical properties. A series of experimentation on different real-life sequential data bases exhibited that the proposed approach substantially secured the sequential pattern mining results not only in terms of extracted patterns but also the support. Furthermore, the results appeared extremely interesting in the case of dense datasets.

Nergiz and Gök (2014) and Nergiz et al. (2013) introduced the hybrid generalizations. It not only performed the generalizations, but also involved the mechanism for data relocation. In data process, the position of certain cells is changed to some populated indistinguishable data cells. The relocation process helped to generate anonymizations of finer granularity and ensured underlying privacy. The data relocation is a trade-off among the utilization and reliability of the data, where the trade-off is controlled by the provider parameter. The results revealed that a small number of relocations could enhance the utility as compared to the heuristic metrics and query answering accuracy. A Hybrid generalizations mechanism to relocate the data is introduced (Nergiz and Gök 2014). In data relocation process, data cells are relocated to certain populated small groups of tuples which remained distinguishable from each other. Again, the data relocation helped to generate anonymizations of finer granularity which ensured the data privacy. It is demonstrated that a small number of relocations could remarkably enhance the utility. New hybrid algorithms can be designed for other privacy metric such as diversity, (α, k)-anonymity or δ-presence. This would be crucial in addressing different types of adversaries. There is also room for improvement of the proposed hybrid algorithms. For example, one can design hybrid algorithms that would theoretically bound to the probability of identification against algorithm-aware adversaries.

Zhang et al. (2013a, 2014a) investigated the issues related to scalability of sub-tree anonymization for huge data storage on cloud. They developed a hybrid approach along with Top-Down Specialization (TDS) and Bottom-Up Generalization (BUG) techniques. In this method, one of the two components is selected automatically by comparing K-anonymity parameter with workload balancing point which is defined by the clients. Both TDS and BUG are obtained in a scalable way via a series of deliberately designed Map Reduce jobs. Based on the contributions herein, it is worth exploring the next step on scalable privacy preservation aware analysis and scheduling on large-scale datasets. Later, Zhang et al. (2014b) introduced a two-phase TDS technique based on Map Reduce on cloud. In the first phase, the data sets are anonymized and partitioned in parallel and intermediate results are generated. In the second phase, these intermediate results are aggregated for further anonymization to produce consistent K-anonymous datasets. The Map Reduce on cloud is employed for data anonymization and a group of data is designed deliberately to concretely achieve the specific computation in a scalable way. The results from the implementation of this method on real-world datasets displayed that the presence of scalability and competence of TDS made the performance much better than existing methods. They have presented an efficient quasi-identifier index based technique to preserve the privacy over incremental datasets on cloud. In the proposed technique, QI-groups (QI: quasi-identifier) are listed using domain values in the current generalization level, which allowed the access only to a small portion of records in any database rather than admittance to the whole data base (Zhang et al. 2013b, c). In addition, Ding et al. 2013) introduced a distributed anonymization protocol for privacy-preserving data publishing from multiple data providers in a cloud system. Their method performed a personalized anonymization to satisfy every data provider’s requirements and the union formed a global anonymization to be published. They also presented a new anonymization algorithm using R-tree index structure.

## Shortcomings of PPDM methods

Currently, several data mining techniques are available to protect the privacy. Broadly, the privacy preserving techniques are classified according to data distribution, data distortion, data mining algorithms, anonymization, data or rules hiding, and privacy protection. Table 1 summarizes different techniques applied to secure data mining privacy. Intensive research findings over the decades revealed that the existing privacy preserving data mining search approaches are still suffer from major incompleteness including the distributed clients’ data to multi semi honest providers, the overhead of computing global mining, incremental data privacy issue in cloud computing, integrity of mining result, utility of data, scalability and overhead performance. Thus, a strong, efficient, and scalable model is essential to surmount these shortcomings. Furthermore, proper anonymization of data is needed to protect the privacy of each client prior to publish. The connection between personal data and personal identification should be vanished. Such an anonymization must not only satisfy underlying privacy requirements but also safeguard the utility of the data.

**Table 1:** Description of PPDM methods

PPDM methods	Description
Data distribution	May contain vertically or horizontally partitioned data
Data distortion	Contain perturbation, blocking, aggregation or merging, swapping and sampling
Data mining algorithms	Encloses classification mining, association rule mining, clustering, and Bayesian networks etc
Data or rules hidden	Denotes to hide main data or rules of innovative data
K-anonymity	Achieve the anonymization
L-diverse	Keeps the least group size of K, and maintains the diversity of the sensitive attributes
Taxonomy Tree	Attributes the generalization to limit the information leakage
Randomization	An un-sophisticated and valuable technique to hide the individual data in PPDM
Privacy protection	Protects the privacy, it should adapt data carefully to attain optimum data utility

Undoubtedly, K-anonymity is an effective method of privacy protection in data mining. However, several demonstrated that the data processed by this method often failed to overcome some attacks and are susceptible to internet phishing. Consequently, the future privacy preserving data mining based K-anonymity needs an advance data infrastructure to support the combination of present data functionality. This would definitely fulfil the requirements of different kinds of clients and communities. Although the present search algorithms are able to speed up the retrieval process, but they do not scale up to large volume of data because of the linear increase of response time with the amount of the searched datasets. The proposed techniques for the searching of distributed large data among many cloud providers must possess the ability to preserve privacy, must be scalable, efficient, compatible and good for utility as well as integrity. Table 2 enlists some relevant studies on privacy preserving data mining as well as their notable merits and de-merits. Table 3 outline the categorization of current studies.

**Table 2:** Relevant literatures on PPDM in terms of their merits and de-merits

References	PPDM, PPDM based on data distortion, data mining, outsourced data mining, distributed and anonymity method	Merits and de-merits	Parameters
Matwin (2013)	Surveyed the existing privacy-preserving data mining methods	Analyzed the methods	PPDM
Vatsalan et al. (2013)	Presented methods that permitted the linking of databases between organizations and preserved the privacy of these data	Presented taxonomy of PPRL techniques	PPDM
Qi and Zong (2012)	Stated methods of data mining for privacy protection	Classified PPDM methods	PPDM
Raju et al. (2009)	Apply homomorphic encryption on multiply protocol	Possible influence in many applications	PPDM
Malina and Hajny (2013), Sachan et al. (2013)	Analyzed current privacy preserving solutions for cloud services and outlined their solution based on advanced cryptographic components	Outputted the experimental results and compared the performance with related solutions	PPDM
Mukkamala and Ashok (2011)	Compared a set of fuzzy based on mapping methods	Combined the multiple practical values of a data item into a single value	PPDM
Kamakshi (2012)	Distortion method, A novel idea to identify the sensitive attributes dynamically	The data is modified be retaining the original properties of the data	Privacy
Zhang et al. (2012a)	Distortion method, proposed HPNGS	Reduced the noise requests over	Privacy and utility
Zhang et al. (2012b)	Distortion method, Proposed a novel APNGS	Improved the effectiveness of privacy protection on noise obfuscation in terms of association probabilities Extra cost in comparison to existing representative strategies is the main demerit	Privacy
Li et al. (2009a)	Distortion method, proposed anonymous perturbation method	Low costs with a high strength	Privacy
Kamakshi and Babu (2010)	Distortion method, proposed model include three parts that are data centers, clients, and database	Customers and their sits database role could be interchangeable	Privacy
Islam and Brankovic (2011)	Distortion method, introduced a framework that incorporates several novel techniques to perturb all attributes of a data set	Effective in preserving original patterns in a perturbed data set	Privacy
Wang and Lee (2008)	Distortion method, proposed an approach to avoid Forward-Inference Attacks, generated by the sanitization process	Restricted Forward-Inference Attacks	Privacy
Shrivastava et al. (2011)	Data mining algorithms, Proposed an improved distortion technique for privacy preserving frequent item-set mining	Enhanced the performance of the algorithm by reducing the disk access time	Privacy and performance
Vijayarani et al. (2010a)	Data mining algorithms, introduced various communities	Focused on importance of association rule	Privacy
Aggarwal and Yu (2008)	Stated that support and confidence are considered the two significant measures within association rule mining	Explained the basic elements of association rule	PPDM
Belwal et al. (2013)	Data mining algorithms, proposed the basis of reduction of support and confidence of sensitive rules	Hided any desired sensitive association rule without any side effect Hidden only the rule that has single sensitive item on the left side is disadvantageous	PPDM
Jain et al. (2011)	Data mining algorithms, proposed a new algorithm that increases and decreases the support of the left side and right side item of hide association rule	Made minimum modification to the data entries to hide a set of rules with lesser CPU time than the previous work	Privacy
Naeem et al. (2010)	Data mining algorithms, proposed an architecture which hides the restricted association rules with the complete removal of the known side effects like the generation of unwanted, non-genuine association rules while yielding no hiding failure	Used other standard statistical measures instead of conventional framework of support and confidence to generate association rules	Privacy
Li and Liu (2009)	Data mining algorithms, Proposed DDIL based on data disturbance and inquiry limitation	Effective, good privacy and accuracy Restriction with random parameters is disadvantageous	Privacy
Weng et al. (2008)	Data mining algorithms, FHSAR Fast Hiding Sensitive Association Rules (SAR) algorithm	Adv. hiding sensitive association rules with limited side effects	Privacy
Dehkordi et al. (2009)	Data mining algorithms, proposed method for hiding sensitive association rules by depending on the concept of genetic algorithms	Offered security as well as keeping the utility	Security and Utility
Gkoulalas-Divanis and Verykios (2009)	Data mining algorithms, proposed a novel approach that offers best solution to hide sensitive frequent item sets	Provided effective solution to hide sensitive frequent item sets	Privacy and efficiency
Li et al. (2009b)	Data mining algorithms, introduced a new algorithm for sanitizing a transactional database	Selection of victim-items with no affection to the non-sensitive patterns is disadvantageous	Privacy
Kasthuri and Meyyappan (2013)	Data mining algorithms, proposed a new method to detect the sensitive items for hiding sensitive association rules	Found the frequent item sets and generates the association rules	Privacy
Quoc et al. (2013)	Data mining algorithms, proposed a heuristic algorithm to hide a set of sensitive association rules using the distortion technique	Specified the victim item and minimum number of transactions	Privacy
Domadiya and Rao (2013)	Data mining algorithms, proposed MDSRRC	Highly efficient and maintains database quality	Privacy, efficiency and quality
Xiong et al. (2006)	Data mining algorithms, used k as the closet neighbor classification technique based on SMC techniques	Balance in accuracy, performance, and privacy protection	Privacy and accuracy.
Singh et al. (2010)	Data mining algorithms, attempted providing a simple and efficient privacy preserving classification for cloud data	Facilitated computing local neighbors at each node in the cloud in a secure way and classifies the unseen records using weighted k-NN classification approach	Privacy
Baotou (2010)	Data mining algorithms, proposed an effective algorithm depending on random perturbation matrix	Enhanced privacy protection and the accuracy	Privacy and accuracy
Vaidya et al. 2008)	Data mining algorithms developed an approach for vertically partitioned mining data	Modified and extended to a variety of data mining applications as decision trees	Privacy and efficiency
Kantarcıoglu and Vaidya (2003)	Data mining algorithms, discussed the use of secure logarithm and summation, where the distributed naive Bayes classifier can be determined securely	Supported the concept that few useful secure protocols facilitated the secure deployment of different types of distributed data mining algorithms	Privacy and accuracy
Sathiyapriya and Sadasivam (2013)	Data mining algorithms, a classification of privacy preserving techniques	The optimal sanitization is proved to be NP-Hard and always there is a trade-off between privacy and accuracy is the notable de-merit	Privacy
Yi and Zhang (2013)	Data mining algorithms, applied k-means clustering on vertically partitioned data	Did not apply any secure two-party computation algorithm is the demerit	Privacy and security
Raghuram and Gyani (2012)	Data mining algorithms, proposed an associative classification model	Accuracy is tested	Privacy
Lin and Lo (2013)	Data mining algorithms, proposed a set of algorithms, containing EWS algorithm, ROD algorithm, SSWS algorithm and the PSWS algorithm	Delivered excellent performance with respect to scalability and execution time	Privacy, scalability and execution time
Harnsamut and Natwichai (2008)	Data mining algorithms, proposed a novel heuristic algorithm to preserve the privacy and maintain the data quality	Efficient and highly effective	Privacy and efficient
Seisungsittisunti and Natwichai (2011)	Data mining algorithms, proposed an incremental polynomial- time algorithm to transform the data to meet a privacy standard	Efficient in every problem setting	Privacy and efficient
Giannotti et al. (2013)	Outsourced data mining, proposed model based on background knowledge of attack	Strong defense against an attack They do not deal with other attack is the demerit	PPDM
Worku et al. (2014)	Outsourced data mining, improved their method by minimizing bilinear mapping	Secured and efficient The demerit is it is not wholly active	PPDM
Arunadevi and Anuradha (2014)	Outsourced data mining, proposed an attack model based on the basic assumption	Improved the security of the system	PPDM
Lai et al. (2014)	Outsourced data mining, proposed the first semantically secure solution for outsourcing association rule mining with data privacy	The demerit is it is non-deterministic and secure against an adversary at cloud servers	PPDM
Kerschbaum and Julien (2008)	Outsourced data mining, proposed a searchable encryption scheme for outsourcing data analytics	Secured	PPDM
Ying-hua et al. (2011)	Distributed, survey on the distributed privacy preserving data mining (DPPDM)	Surveyed on the DPPDM	PPDM
Li (2013)	Distributed, designed, and analyzed a symmetric-key based privacy- preserving scheme for mining support counts	Effective in detecting misbehaving nodes and increasing average throughput in the whole network	Privacy
Dev et al. (2012)	Distributed, combining categorization, fragmentation and distribution, prevents data mining by maintaining privacy levels, splitting data into chunks and storing these chunks of data to appropriate cloud providers	Provided an effective way to protect privacy from mining based attacks It introduced performance overhead as demerit	Privacy
Tassa (2014)	Distributed, proposed a protocol based on association rules in horizontally distributed databases	Devised an effective protocol for disparity verifications is disadvantageous	Privacy, accuracy and efficiency
Chan and Keng (2013)	Distributed, proposed a distributed architecture for privacy preserving outsourcing of association rules mining	Computational and storage overheads are significantly reduced in such a scheme	Privacy
Dong and Kresman (2009)	Distributed, focused on the linking between distributed data mining	It is simple to implement with least computing requirements	Privacy
Aggarwal et al. (2005)	Distributed, have discussed the developed techniques such as services based on data encryption, causing a large overhead in query processing and proposed a new distributed framework to enable privacy-preservation for the outsourced storage of data	A new definition for privacy has been demonstrated based on hiding sets of attribute values and it also discussed how proposed decomposition approaches help to achieve privacy, and identify the best privacy-preserving decomposition technique	Privacy
Xu and Yi (2011)	Distributed, proposed taxonomy to categorize those PPDDM protocols into important categories	High performance of these protocols	Privacy
Inan and Saygin (2010)	Distributed, proposed a method which constructs different matrix in the horizontal distributed data mining	Provided different comparison function for either character or numerical data	Privacy
Nanavati and Jinwala (2012)	Distributed, proposed techniques that protect privacy for global and partial cycles in a distributed data	Distinguished global cycles in a cooperative setup	Privacy
Agrawal and Srikant (2000)	Distributed, have developed a uniform randomization method based association rule for the categorical datasets	The data reassembled is sanitized knowledge based	Privacy
Wang et al. (2010)	Distributed, proposed an enhanced algorithm (PPFDM)	An effective and appropriate for the practical application fields	Privacy
Nguyen et al. (2012)	Distributed, Proposed Enhanced Scheme (EMHS)	Performance is better than MHS in specific databases	Privacy
Om Kumar et al. (2013)	Distributed, used WEKA to predict the patterns in a single cloud and by using cloud data distributor with a secure distributed approach	An effective solution that prevents such mining attacks on cloud thus making the cloud a secure platform for service and storage	Privacy
Mokeddem and Belbachir (2010)	Distributed, proposed model allowing the class association rules detection in a shared-nothing architecture	Created classification rules in a parallel setting	Privacy
Ibrahim et al. (2012)	Distributed, presented a practical cryptographic method to compute the KNN classification problem	Demonstrated that accuracy of the proposed work is the same as that of a naive scheme without security	Privacy
Patel et al. (2012)	Distributed, stated an effective algorithm to preserve privacy of distributed K-Means clustering	Faster than other algorithms and it is more appropriate for huge datasets in practical scenario	privacy
Kumbhar and Kharat (2012)	Distributed, analyzed different methods for PPARM	Studied the methods that depended on association rules mining on distributed dataset	Privacy
Nix et al. (2012)	Distributed, implemented two sketching protocols for the scalar (dot) product of two vectors which can be used as sub-protocols in larger data mining tasks	Accuracy and efficiency results through extensive experimentation	Privacy, accuracy and efficiency
Keshavamurthy et al. (2013)	Distributed, proved approach of Genetic Algorithm (GA) has two potential advantages comparison with traditional frequent pattern mining algorithm	The fitness function of GA plays an important role, and the convergence of search space is directly proportionate to the effectiveness of fitness function The GA could result in duplicate formation in its successive generations is a de-merit	Privacy
Loukides et al. (2012), Machanavajjhala et al. (2007)	Anonymity, proposed a novel approach that fulfils utility of data requirements	Effective	Privacy and utility
Wang et al. (2004)	Anonymity, have studied data mining as approach used for data masking, known as data mining-based privacy protection	Two key factors, quality and scalability has been focused specifically is advantageous	Privacy, quality, and scalability
Friedman et al. (2008), Loukides and Gkoulalas-divanis (2012)	Anonymity, presented definitions of k-anonymity	It could be used in many data mining algorithms	Privacy
Ciriani et al. (2008)	Anonymity, presented the possible threats to K-anonymity and categorized two main approaches for merging K- anonymity in data mining	Discussed different methods that could be applied to detect K-anonymity violations	Privacy
He et al (2011), Friedman et al. (2008)	Anonymity, proposed an algorithm which is based on clustering to produce a utility-friendly anonymized version of micro data	Utility is improved by their approach	Privacy and utility
Patil and Patankar (2013), He et al. (2011)	Anonymity, analyzed existing K-anonymity model and its applications	Analyzed current K-anonymity model	Privacy
Zhu and Chen (2012), Patil and Patankar (2013)	Anonymity, studied K-anonymity model	Surveyed K-anonymity model	Privacy
Soodejani et al. (2012), Zhu and Chen (2012)	Anonymity, employed a version of the chase, called standard chase	Provided a stronger privacy model for the proposed method and can be valuable	Privacy
Karim et al. (2012), Soodejani et al. (2012)	Anonymity, proposed a numerical method to mine maximal frequent patterns with privacy preserving capability	An efficient data transformation technique, a novel encoded and compressed lattice structure, and MFPM algorithm	Privacy
Loukides et al. (2012), Karim et al. (2012)	Anonymity, proposed a rule-based privacy model that allows data publishers to express fine-grained protection requirements for both identity and sensitive information disclosure	Outperformed the state-of-the-art in terms of retaining data utility, while achieving good protection	Privacy, utility and scalability
Vijayarani et al. (2010a, b), Loukides et al. (2012)	K-anonymity has been studied as an interesting approach to protect micro data related to public or semi-public sectors from linking attacks	Proposed novel approach	Privacy
Nergiz et al. (2009), Xu and Yi (2011)	Anonymity, proposed new clustering algorithms to achieve multi relational anonymity	Provided utility of data and efficiency	Utility, effectiveness and efficiency
Tai et al. (2013), Vijayarani et al. (2010b)	Anonymity, proposed a Distributed *k*-support Noise Taxonomy tree algorithm, abbreviated as DKNT	Achieved good protection and better computation efficiency, as compared to the computation efficiency on single machine	Privacy and efficiency
Tai et al. (2010, 2013)	Anonymity, introduce a pseudo taxonomy tree and have the third party mine the generalized frequent item-sets instead	Achieved very good privacy protection with moderate storage overhead	Privacy
Pan et al. (2012), Tai et al. (2010)	Anonymity, had analyzed and performed a comparison for the present developed K-anonymity models and its applications	Enhanced K -anonymity and improve it	Privacy
Deivanai et al. (2011), Pan et al. (2012)	Anonymity, proposed novel method named kactus	Accuracy is better than other methods based on K -anonymity	Privacy and accuracy
Monreale et al. (2014), Deivanai et al. (2011)	Anonymity, a new definition of K-anonymity for personal sequential data which provides an effective privacy protection model is introduced	Results are extremely interesting in the case of dense datasets	Privacy
Nergiz et al. (2013), Monreale et al. (2014)	Anonymity, the hybrid generalizations with data relocation	Increased the utility of data	Privacy and utility
Zhang et al. (2013a, 2014a), Nergiz et al. (2013)	Anonymity, proposed hybrid approach by combining Top-Down Specialization and Bottom-Up Generalization	Improved the scalability and efficiency of TDS	Privacy and scalability
Zhang et al. (2014a)	Anonymity, proposed a highly scalable two-phase TDS approach using Map Reduce on cloud	Scalability and efficiency of TDS are improved significantly over existing approaches	Privacy and scalability
Zhang et al. (2013a, b), Zhang et al. (2014a)	Anonymity, proposed method depends on an efficient quasi-identifier index	Protected privacy when new data is added	Privacy and efficiency
Nergiz and Gök (2014)	Anonymity, Hybrid generalizations	Ensured the utility of data	Privacy and utility
Ding et al. (2013), Zhang et al. (2013c)	Anonymity, have presented a distributed anonymization protocol for privacy-preserving data publishing from multiple data providers in a cloud system	Performed a personalized anonymization to satisfy every data provider’s requirements and the union forms a global anonymization to be published	Privacy

**Table 3:** **C**ategorization of current studies

References	PPDM	Hide association rule	Distortion method	Classification method	Clustering method	Associative classification	Outsource data mining	Association rule	Distributed method	K-anonymity	Parameters
Matwin (2013)	√										Privacy
Vatsalan et al. (2013)	√										Privacy
Qi and Zong (2012)	√										Privacy
Raju et al. (2009)	√										Privacy
Malina and Hajny (2013), Sachan et al. (2013)	√										Privacy
Mukkamala and Ashok (2011)	√	√									Privacy
Weng et al. 2008)		√									Privacy and efficiency
Dehkordi et al. 2009)		√									Security, privacy and utility
Gkoulalas-Divanis and Verykios (2009)		√									Efficiency
Li et al. (2009b)		√									Privacy
Kasthuri and Meyyappan (2013)		√									Privacy
Quoc et al. (2013)		√									Privacy
Domadiya and Rao (2013)		√									Efficient
Kamakshi (2012)			√								Privacy and utility
Zhang et al. (2012a)			√								Privacy
Zhang et al. (2012b)			√								Privacy
Li et al. (2009a)			√								Privacy
Kamakshi and Meyyappan (2010)			√								Privacy
Islam and Brankovic (2011)			√								Privacy
Wang and Lee (2008)			√								Accuracy, efficiency, and privacy
Xiong et al. 2006)				√							Privacy
Singh et al. (2010)				√							Privacy and efficiency
Baotou (2010)				√							Privacy
Vaidya et al. (2008)				√							Privacy
Kantarcıoglu and Vaidya (2003)				√							Privacy and accuracy
Sathiyapriya and Sadasivam (2013)				√							Security and privacy
Yi and Zhang (2013)					√						Accuracy and privacy
Raghuram and Gyani (2012)						√					Performance, scalability and execution time
Lin and Lo (2013)						√					Privacy, efficiency, and effective
Harnsamut and Natwichai (2008)						√					Privacy and efficient
Seisungsittisunti and Natwichai (2011)						√					Privacy
Giannotti et al. (2013)							√				Security and privacy
Worku et al. (2014)							√				Security and privacy
Arunadevi and Anuradha (2014)							√				Security and privacy
Lai et al. (2014)							√				Security and privacy
Kerschbaum and Julien (2008)							√				Privacy and performance
Shrivastava et al. (2011)								√			Efficiency and security
Vijayarani et al. (2010a)								√			Privacy and utility
Aggarwal and Yu (2008)								√			Privacy
Belwal et al. (2013)								√			Privacy
Jain et al. (2011)								√			Privacy
Naeem et al. (2010)								√			Privacy and accuracy
Li and Liu (2009)								√			Efficiency
Ying-hua et al. (2011)									√		Privacy
Li (2013)									√		Privacy
Dev et al. (2012)									√		Privacy
Tassal (2014)									√		Privacy
Chan and Keng (2013)									√		Privacy
Dong and Kresman (2009)									√		Privacy
Aggarwal et al. (2005)									√		Privacy
Xu and Yi (2011)									√		Privacy
Inan and Saygin (2010)									√		Privacy
Nanavati and Jinwala (2012)									√		Privacy
Agrawal and Srikant (2000)									√		Privacy and efficient
Wang et al. (2010)									√		Privacy and performance
Nguyen et al. (2012)									√		Privacy and an effective
Om Kumar et al. (2013)									√		Privacy
Mokeddem and Belbachir (2010)									√		Privacy and accuracy
Ibrahim et al. (2012)									√		Privacy and efficiency
Patel et al. (2012)									√		Privacy
Kumbhar and Kharat (2012)									√		Privacy, accuracy and efficiency
Nix et al. (2012)									√		Privacy
Keshavamurthy et al. (2013)									√		Accuracy and privacy
Wang et al. (2004), Machanavajjhala et al. (2007)										√	Privacy
Loukides et al. (2012), Wang et al. (2004)										√	Privacy, efficiency, accuracy and utility
Friedman et al. (2008), Loukides and Gkoulalas-divanis (2012)										√	Privacy and utility
Ciriani et al. (2008)										√	Privacy
He et al. (2011), Friedman et al. 2008)										√	Privacy and utility
Patil and Patankar (2013), He et al. (2011)										√	quality of anonymity and reduce the information loss
Zhu and Chen (2012), Patil and Patankar 2013)										√	Privacy
Soodejani et al. (2012, Zhu and Chen (2012)										√	Privacy
Karim et al. (2012), Soodejani et al. (2012)										√	Privacy
Loukides et al. (2012), Karim et al. (2012)										√	Privacy
Vijayarani et al. (2010a, b), (Loukides et al. 2012)										√	Privacy
Nergiz et al. (2009), Xu and Yi (2011)										√	Privacy, utility and effectiveness
Tai et al. (2013), Vijayarani et al. (2010b)										√	Privacy and efficiency
Tai et al. (2010, 2013)										√	Privacy
Pan et al. (2012), Tai et al. (2010)										√	Privacy
Deivanai et al. (2011), Pan et al. (2012)										√	Privacy
Monreale et al. (2014), Deivanai et al. (2011)										√	Privacy
Nergiz and Gök (2014), Nergiz et al. (2013), Monreale et al. (2014)										√	Privacy, utility and accuracy
Zhang et al. (2013a, 2014a), Nergiz et al. (2013)										√	Privacy and scalable
Zhang et al. (2014a)										√	Privacy and scalability
Zhang et al. (2013a, b), Zhang et al. (2014a)										√	Privacy, scalability and efficiency
Ding et al. (2013), Zhang et al. (2013c)										√	Privacy

## Conclusion

An inclusive overview on PPDM techniques based on distortion, associative classification, randomization, distribution, and k-anonymization is presented. It is established that PPDM is appeared progressively common due to easy sharing of privacy sensitive data for analysis. The notable advantages and obvious disadvantages of current studies are emphasized. Presently, Big Data are often shared across sectors such as health, military and others, and transverses across Business-to-Businesses, Entities-to-Entities and Government-to-Government. Thus, the preservation of privacy against disclosure and attacks are of critical concern. Several big organizations and governments worldwide being totally dependent on information communications via internet expressed grave concerns over privacy issues. Consequently, the rapid development of IT faced new challenges to PPDM. Data mining possesses being the capability to extract and mine vast sea of interesting patterns or knowledge from a huge amount of data requires absolute security. The main idea of PPDM is to incorporate the traditional data mining techniques in transforming the data to mask sensitive information. The major challenge is to efficiently transform the data and recover its mining outcome from the transformed one. Furthermore, the incompleteness of previous studies indicated forced us to engage in an extensive inspection of the problems of distributed and published data for sharing and mining. Consequently, the overhead for global mining computing, preserving privacy of growing data, the integrity of mining result, the utility of data, the scalability and overhead performance in the context of PPDM are examined. There is an urgent necessity to develop a strong, efficient, and scalable model to surmount these issues. In this regard, we identified the gaps and weaknesses of existing literatures and analyzed them for further significant enhancements, more robust privacy protection, and preservation. This exhaustive and informative review article is hoped to serve as taxonomy for navigating and comprehending the research advancements towards PPDM.
